# Risk factors for postoperative cognitive dysfunction in geriatric trauma: a dual-pathway perspective

**DOI:** 10.3389/fnagi.2026.1841750

**Published:** 2026-07-17

**Authors:** Xiao-dong Wang, Jiao Xue, Ting-ting Qiao, Chang-jiao Yang, Xiao-yan Li, Yi Qiu

**Affiliations:** 1 Department of Anaesthesiology, The Second Affiliated Hospital of Inner Mongolia Medical University, Hohhot, China; 2 College of Humanities and Education, Inner Mongolia Medical University, Hohhot, China

**Keywords:** blood–brain barrier, cognitive reserve, geriatric trauma, neuroinflammation, perioperative management, peripheral stress amplification, postoperative cognitive dysfunction

## Abstract

Postoperative cognitive dysfunction (POCD) is common in geriatric trauma patients and involves complex underlying mechanisms. Most existing studies have focused on isolated risk factors, with limited attention to how these factors interact. In this perspective article, we propose a dual-pathway theoretical model—central nervous system vulnerability and peripheral stress amplification—to reframe the understanding of POCD pathogenesis. In older adults, POCD rarely stems from a single cause. Instead, it arises from a “second hit” process, in which pre-existing central nervous system vulnerabilities (e.g., neurodegenerative pathology and reduced cognitive reserve) interact with perioperative peripheral stressors (e.g., trauma, surgical stress, and anesthetic exposure) through interfaces such as the blood–brain barrier and neuroimmune circuits. Guided by this dual-pathway framework, future research should move from isolated risk factor screening toward dynamic, multidimensional, and individualized risk stratification, and explore sequential intervention strategies that target both pathways.

## 1 Introduction

### 1.1 Clinical challenges of postoperative cognitive dysfunction (POCD) in geriatric trauma

As the global population ages, more older trauma patients undergo surgery (Deiner and Silverstein, 2009). POCD is a common central nervous system complication in this group, marked by declines in attention, memory, and executive function (Newfield, 2009; Wu et al., 2023). The reported incidence of perioperative neurocognitive disorders in older surgical patients varies widely, from about 10% to 40%, depending on surgery type, perioperative stress intensity, and baseline cognitive vulnerability (Arefayne et al., 2023; Zhao et al., 2024). In geriatric trauma populations, this risk may be even higher because trauma-related systemic inflammation, hemodynamic instability, and the urgency of emergency surgery often coexist with pre-existing neurodegenerative and cerebrovascular conditions (Albanese et al., 2022; Subramaniyan and Terrando, 2019). Beyond prolonging hospitalization and increasing long-term mortality, POCD significantly reduces quality of life and frequently leads to dependence on long-term care (Nilsson et al., 2025; Suraarunsumrit et al., 2024). Given its insidious onset and lack of specific treatments, the high incidence and poor prognosis of POCD pose a major clinical challenge in geriatric trauma surgery, placing substantial burdens on health care systems and society (Berger et al., 2015).

### 1.2 Limitations of existing risk factor research

Previous studies have identified multiple risk factors for POCD, including advanced age, low preoperative cognitive reserve, surgical trauma intensity, anesthetic type, perioperative stress, and systemic inflammation (Rundshagen, 2014). However, this body of research remains largely fragmented (Rundshagen, 2014; Tang et al., 2025). First, how these factors interact and relate to one another has not been systematically integrated (Kitthanyateerakul et al., 2024). Second, most analytical models do not account for the unique pathophysiological context of older adults—such as age-related susceptibility to neuroinflammation, multisystem degeneration, and the compounded effects of multiple chronic conditions—as a unified framework (Jia et al., 2023). This “factor-listing” approach limits the development of precise risk prediction models and hinders the translation of mechanistic insights into effective interventions.

### 1.3 Need for a dual-pathway framework

**FIGURE 1:**
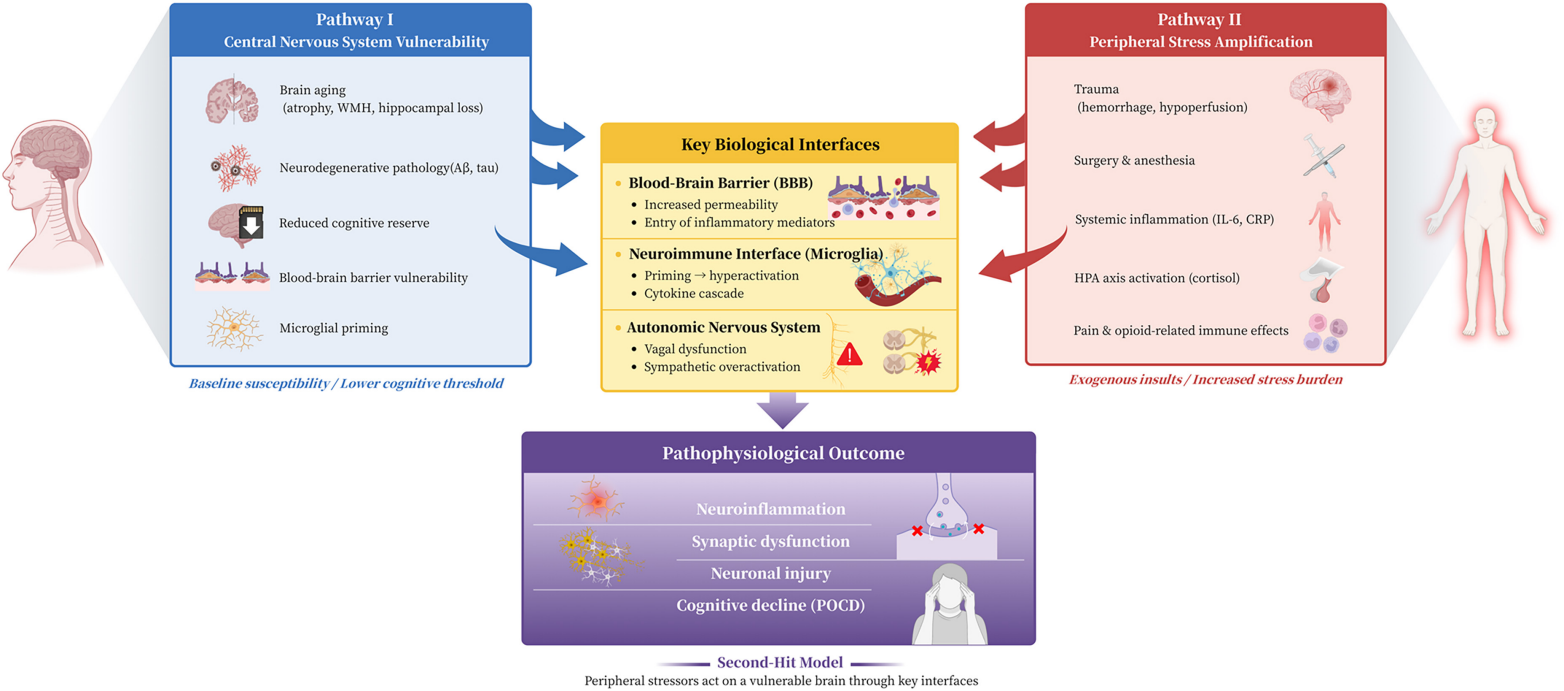
Dual-pathway mechanism underlying POCD in geriatric trauma. The Figure was created using BioRender.com.

To address these limitations, this perspective article proposes a “dual-pathway framework” as an integrated theoretical model. This framework conceptualizes POCD pathogenesis along two interrelated but mechanistically distinct pathways. The first, the “neuroinflammation-dominant pathway,” emphasizes how surgery-induced systemic inflammation is transmitted to the CNS and leads to microglial overactivation, which impairs synaptic plasticity (Skvarc et al., 2018; Subramaniyan and Terrando, 2019). The second, the “neural reserve depletion pathway,” focuses on how aging and comorbidities reduce cognitive reserve, impair cerebrovascular function, and exhaust the compensatory capacity of neural networks, making older individuals especially vulnerable to exceeding cognitive thresholds under perioperative stress (Deiner and Silverstein, 2009; Stern, 2012). These two pathways interact synergistically across molecular, cellular, and circuit levels, jointly determining the onset and progression of POCD (Evered et al., 2018; Figure 1).

### 1.4 Article structure and perspective

This perspective article first examines the theoretical foundations and empirical evidence underlying the dual-pathway framework. It then details the mechanistic features of each pathway and their specific manifestations in the geriatric trauma population, followed by a discussion of their potential interactions and clinical implications. Our central perspective is that advancing the understanding of POCD requires shifting from a “factor-summation” approach to a “mechanism-integration” perspective—one that places POCD within the interactive context of aging and perioperative stress. Such an integrated framework is essential to overcoming current research bottlenecks and providing a theoretical basis for precision strategies in perioperative brain protection.

## 2 Pathway I: central nervous system vulnerability—endogenous susceptibility basis

This section addresses the intrinsic characteristics of the aging central nervous system that predate surgery and trauma. These features determine an individual’s baseline sensitivity to perioperative insults and constitute the susceptibility foundation for POCD.

### 2.1 Structural and functional brain aging

Normal aging entails substantial structural and functional brain alterations (Habes et al., 2016). Neuroimaging studies frequently reveal preoperative changes in older adults, including white matter hyperintensities, global brain atrophy, and reduced hippocampal volume (Habes et al., 2016). These findings reflect neuronal loss and diminished white matter integrity (Liu et al., 2017). Aging of the neurovascular unit is another hallmark of brain aging (Banks et al., 2021), characterized by increased blood–brain barrier (BBB) permeability, attenuated vascular reactivity, and impaired perivascular clearance under physiological conditions (Hergert et al., 2024). Such changes render the aged brain more vulnerable to microenvironmental disturbances when confronted with surgical trauma and anesthetic exposure (Banks et al., 2021; Hergert et al., 2024). In addition to structural and vascular aging, older adults often exhibit a decline in autonomic regulation (Jiang et al., 2022; Olivieri et al., 2024). Specifically, impairment of the vagus nerve–mediated cholinergic anti-inflammatory pathway—manifested by reduced acetylcholine release and weakened α7 nicotinic acetylcholine receptor signaling—diminishes endogenous anti-inflammatory control, thereby heightening susceptibility to excessive systemic and neuroinflammatory responses during perioperative stress (Echeverria et al., 2023; Jiang et al., 2022; Zhang et al., 2023; Zorbaz et al., 2022).

### 2.2 Pathological comorbidities and subclinical neurodegeneration

Beyond physiological aging, older patients frequently harbor subclinical or overt neurodegenerative pathology. Alzheimer’s disease–related changes, such as amyloid-β deposition and hyperphosphorylated tau accumulation, are common even in the absence of dementia (Terrando et al., 2011). This pathological burden sensitizes the brain to perioperative inflammatory stress, amplifies neuroinflammatory responses, and accelerates cognitive decline (Liu et al., 2022; Subramaniyan and Terrando, 2019). Mechanistically, amyloid-β accumulation promotes a proinflammatory microglial phenotype by engaging pattern-recognition receptors and inflammasome-related signaling, thereby enhancing the release of interleukin-1β, tumor necrosis factor-α, and reactive oxygen species under perioperative conditions (Seplovich et al., 2025; Wang et al., 2020). Concurrently, hyperphosphorylated tau disrupts synaptic integrity and mitochondrial function, rendering neurons more vulnerable to inflammatory and metabolic insults (Seplovich et al., 2025; Wu et al., 2021). A bidirectional pathophysiological relationship has been identified: experimental evidence shows that surgery-induced systemic inflammation can, in turn, accelerate tau phosphorylation and exacerbate amyloid-β-related neurotoxicity, perpetuating a vicious cycle that drives persistent postoperative cognitive decline in susceptible older individuals (Chen et al., 2025; Yu et al., 2021). Concomitantly, cerebrovascular conditions—such as microinfarcts, atherosclerosis, and cerebral small vessel disease—impair cerebral autoregulation (Alam et al., 2018; Ren et al., 2025). This disruption compromises the dynamic balance between oxygen supply and metabolic demand under stress (Ren et al., 2025), further reducing the brain’s resilience to perioperative insults.

### 2.3 Cognitive reserve and neural compensatory mechanisms

Cognitive reserve denotes the brain’s capacity to sustain cognitive function despite neuropathological damage (Stern, 2012). Proxy indicators such as years of education, occupational attainment, and participation in cognitively stimulating activities are commonly used to estimate cognitive reserve (Stern, 2012). Epidemiological evidence demonstrates a clear inverse association between greater cognitive reserve and the incidence of POCD (Feinkohl et al., 2017). At the network level, aging is linked to diminished functional connectivity within resting-state networks, particularly the default mode network (Damoiseaux et al., 2008), manifesting as reduced intra-network connectivity and increased inter-network dedifferentiation (Chan et al., 2014). This loss of network flexibility impairs the brain’s ability to reconfigure functional connections and maintain cognitive performance under stress, thereby heightening susceptibility to perioperative cognitive decompensation (Cabeza et al., 2018).

## 3 Pathway II: peripheral stress amplification—exogenous triggers and aggravating factors

This section examines the systemic stress responses induced by traumatic injury and perioperative management, how these responses are converted into central nervous system injury signals, and how they initiate or exacerbate POCD in the context of pre-existing vulnerability.

### 3.1 Dual insults of trauma itself

Trauma imposes a dual insult on older patients. The first component is acute blood loss and hypoperfusion. Traumatic hemorrhage can sharply reduce effective circulating volume and lower cerebral perfusion pressure, markedly increasing the risk of secondary cerebral ischemia and hypoxia (Karagianni et al., 2025). This risk is especially pronounced in older individuals with pre-existing cerebral small vessel disease or impaired autoregulation, in whom even mild hemodynamic disturbances may produce occult brain injury (Iadecola, 2013). The second component is the systemic inflammatory response syndrome triggered by tissue damage (Lord et al., 2014). Extensive soft tissue contusion or bone fracture activates the innate immune system, releasing large quantities of proinflammatory cytokines and establishing a systemic inflammatory state that primes the central nervous system for a secondary insult (Dobson et al., 2022).

### 3.2 Neurotoxicity and immunomodulation associated with anesthesia and surgery

Anesthesia and surgical procedures act as independent stressors. Regarding anesthetics, volatile agents and certain intravenous drugs may exert neurotoxic effects on the aged brain (Safavynia and Goldstein, 2019; Wu et al., 2025), involving mitochondrial dysfunction, neuronal apoptosis, and suppressed neurogenesis (Safavynia and Goldstein, 2019; Wu et al., 2025). Preclinical studies have identified specific underlying mechanisms, demonstrating that volatile anesthetics such as isoflurane and sevoflurane can induce calcium dysregulation, mitochondrial oxidative stress, and activation of apoptosis-related signaling pathways in aged neurons (Osman et al., 2023; Qin and Deng, 2026). In vulnerable aging brains, anesthetic exposure has also been associated with increased amyloid-β production, tau hyperphosphorylation, impaired hippocampal neurogenesis, and disrupted synaptic plasticity (Horan et al., 2023; Terrando et al., 2011). Although the clinical translation of these findings remains under investigation, the convergent evidence suggests that anesthetic-associated neurotoxicity may interact synergistically with pre-existing neurodegenerative vulnerability, thereby worsening perioperative neurocognitive impairment (Hussain et al., 2014; Qin and Deng, 2026). Regarding surgical factors, surgical trauma elicits sterile inflammation by releasing damage-associated molecular patterns such as high-mobility group box 1 and activating the complement cascade (He et al., 2012; Saxena et al., 2021; Terrando et al., 2016). These inflammatory signals are subsequently transmitted to the central nervous system through a compromised BBB or via neural–immune pathways (Safavynia and Goldstein, 2019). Collectively, these factors increase the inflammatory burden on the central nervous system.

### 3.3 Perioperative stress–immune–metabolic dysregulation

The perioperative stress response extends beyond inflammatory pathways to encompass widespread neuroendocrine and metabolic disturbances. The hypothalamic–pituitary–adrenal axis becomes overactivated following trauma and surgical stimulation, leading to abnormally elevated cortisol levels or disruption of its normal circadian rhythm (Desborough, 2000). Prolonged or excessive glucocorticoid exposure can directly damage brain regions subserving cognition by inhibiting hippocampal neuronal plasticity, promoting dendritic atrophy, and reducing neurogenesis (McEwen, 1998). In addition, postoperative pain acts as a persistent stressor. Persistent postoperative pain activates peripheral and central inflammatory signaling pathways, thereby contributing to neurocognitive dysfunction (Khaled et al., 2025; Qin and Deng, 2026). Sustained nociceptive stimulation increases the release of proinflammatory mediators—including interleukin-1β, interleukin-6, prostaglandins, and tumor necrosis factor-α—which in turn promote microglial activation and synaptic dysfunction in cognition-related brain regions (Kong et al., 2024). Concurrently, chronic pain-related stress disrupts hippocampal neuroplasticity, impairs long-term potentiation, and alters functional connectivity within memory-related neural networks (Liu et al., 2024; Meng et al., 2025). Thus, inadequate postoperative pain control may act not only as a physiological stressor but also as a neuroinflammatory amplifier, directly contributing to the development of postoperative cognitive dysfunction. Opioid analgesics, although effective for pain relief, possess immunomodulatory properties that may suppress cellular immunity and interfere with the resolution of inflammation (Ji et al., 2018; Sacerdote, 2006), creating a complex balance between adequate analgesia and appropriate immune regulation. The combined dysregulation of the stress–immune–metabolic network represents the peripheral mechanism that amplifies central nervous system injury (Cibelli et al., 2010).

## 4 Interaction mechanisms of dual pathways: from parallelism to convergence

This section forms the core of the present perspective article. It demonstrates that central nervous system vulnerability (Pathway I) and peripheral stress amplification (Pathway II) do not operate independently. Rather, they interact through multiple biological interfaces to jointly drive the onset and progression of POCD.

### 4.1 Interface 1: the BBB—coupling vulnerability with permeability

The BBB is the critical interface between the central nervous system and the peripheral circulation. Age-related BBB vulnerability, as delineated in Pathway I—including reduced expression of tight junction proteins, diminished pericyte coverage, and structural alterations of the basement membrane—provides the structural basis for peripheral inflammatory signals to enter the central nervous system (Montagne et al., 2015; Sweeney et al., 2018). Superimposed on this susceptibility, the systemic inflammatory response following trauma (Pathway II) further compromises BBB integrity (Varatharaj and Galea, 2017). Proinflammatory cytokines and damage-associated molecular patterns can downregulate tight junction proteins such as claudin-5 and occludin, increase vascular permeability, and establish a vicious cycle of vulnerability substrate and inflammatory injury (Erickson and Banks, 2019; Yang et al., 2017). This cycle facilitates the entry of peripheral inflammatory mediators into the brain parenchyma.

### 4.2 Interface 2: neuroimmune interactions—microglial priming and hyperactivation

Microglia, the intrinsic immune cells of the central nervous system, play a central role in the interplay between the two pathways. Aging-related changes accumulating through Pathway I—including amyloid deposition, tau pathology, and chronic low-grade inflammation—shift microglia into a primed state (Cunningham, 2013; Perry and Holmes, 2014), characterized by morphological alterations, elevated basal cytokine expression, and a lowered threshold for immune activation. When peripheral stress signals from Pathway II (such as surgical trauma or systemic inflammation) reach the central nervous system via the BBB or other routes, primed microglia mount an exaggerated response (Cunningham, 2013; Terrando et al., 2010), releasing substantial quantities of proinflammatory cytokines (e.g., interleukin-1β, tumor necrosis factor-α) and generating a central inflammatory storm (Terrando et al., 2010). This storm can directly impair cognitive function by disrupting synaptic plasticity, particularly by inhibiting long-term potentiation in neural circuits essential for learning and memory (Yirmiya and Goshen, 2011).

### 4.3 Interface 3: the autonomic nervous system—an amplifier of stress signals

The autonomic nervous system serves a key regulatory role in the bidirectional transmission of central and peripheral stress signals. The vagus nerve–mediated cholinergic anti-inflammatory pathway constitutes an intrinsic mechanism for limiting excessive inflammation (Pavlov and Tracey, 2012; Tracey, 2002). As discussed in Pathway I, the age-related decline of this regulatory pathway weakens endogenous anti-inflammatory capacity and heightens vulnerability to excessive neuroinflammatory responses under perioperative stress. Concurrently, sympathetic overactivation can lead to reduced heart rate variability, increased blood pressure fluctuations, and impaired cerebral autoregulation (Thayer et al., 2010). These changes further destabilize cerebral perfusion, rendering vulnerable brain regions more susceptible to ischemic and hypoxic injury under stress (Iadecola, 2013). Thus, within the dual-pathway framework, the autonomic nervous system functions not only as a physiological regulator but also as a dynamic interface that amplifies the transmission of stress signals between peripheral inflammatory responses and central nervous system vulnerability.

### 4.4 Integrated model

Bringing together the aforementioned interaction interfaces, an integrated model of POCD pathogenesis can be proposed. Central nervous system vulnerability (Pathway I) establishes the background susceptibility state, characterized by compromised BBB integrity, primed microglia, and reduced autonomic regulatory capacity (Skvarc et al., 2018; Subramaniyan and Terrando, 2019). Peripheral stress amplification (Pathway II) provides the triggering force, delivering multidimensional insults through systemic inflammation, hemodynamic fluctuations, and neuroendocrine disturbances (Evered and Silbert, 2018; Safavynia and Goldstein, 2019). The two pathways converge through three key interfaces—the BBB, microglia, and the autonomic nervous system—ultimately leading to synaptic dysfunction, neuronal injury, and clinically evident cognitive decline (Bowman et al., 2018). This model emphasizes the mechanistic progression from parallelism to convergence and offers an integrated theoretical framework for the prevention and intervention of POCD (Supplementary Table 1).

## 5 Clinical implications and future directions: precision management based on the dual-pathway framework

Building on the dual-pathway interaction model, the clinical management of POCD can shift from the identification of isolated risk factors toward mechanism-guided precision management. This section focuses on risk stratification, intervention strategies, and future research priorities.

### 5.1 Risk stratification: from static assessment to dynamic monitoring

Traditional risk prediction models for POCD rely primarily on static preoperative variables and often fail to capture dynamic perioperative changes (Evered et al., 2018). The dual-pathway framework provides a more comprehensive approach to risk stratification. During the preoperative phase, assessment should emphasize central nervous system vulnerability (Pathway I) (Silbert et al., 2015). Neuroimaging techniques may be used to quantify white matter hyperintensity burden, cerebral atrophy, and hippocampal volume. In addition, emerging biomarkers, including serum neurofilament light chain and glial fibrillary acidic protein, may help reflect neuroaxonal injury and astrocyte activation, respectively (Safavynia and Goldstein, 2019).

**FIGURE 2:**
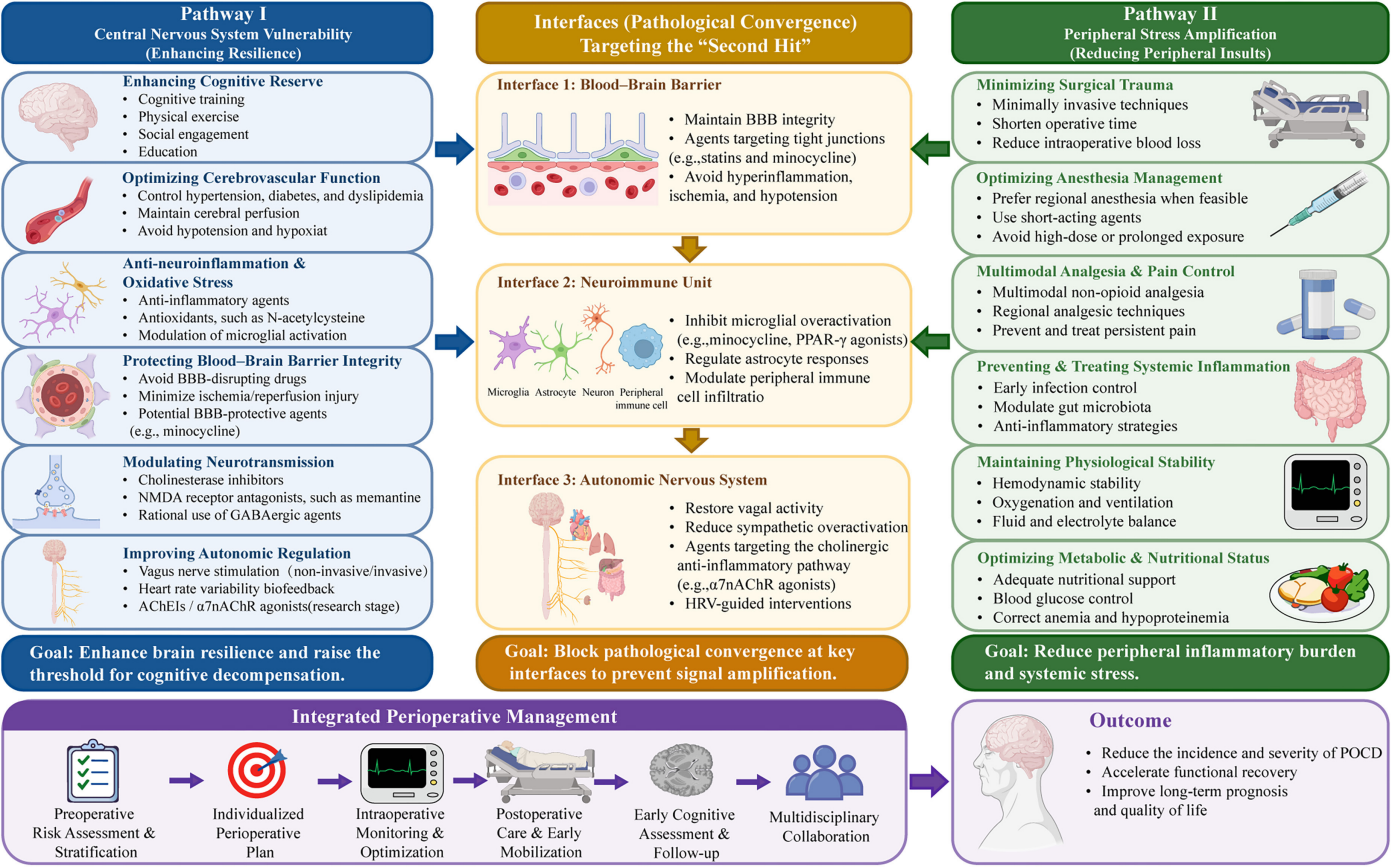
Dual-pathway framework of POCD and comprehensive intervention strategies.

During the perioperative period, dynamic monitoring should focus on the degree of peripheral stress amplification (Pathway II). This includes serial assessment of systemic inflammatory markers, such as interleukin-6 and C-reactive protein, as well as regional cerebral oxygen saturation, which may provide real-time information regarding inflammatory burden and cerebral oxygen balance (Deschamps et al., 2016; Vacas et al., 2013). Integrating baseline vulnerability assessment with dynamic stress monitoring may improve individualized risk stratification and facilitate the early identification of high-risk patients (Figure 2).

### 5.2 Redefining intervention strategies

The dual-pathway framework provides a hierarchical strategy for POCD prevention and management. For Pathway I (enhancing resilience), the primary objective is to improve central nervous system reserve capacity and tolerance to perioperative stress. Potential approaches include preoperative cognitive prehabilitation, optimized control of cerebrovascular risk factors such as hypertension and hyperglycemia, and interventions aimed at slowing subclinical neurodegenerative progression (Evered et al., 2018; Humeidan et al., 2021). For Pathway II (reducing peripheral insult), the focus is to minimize the intensity and duration of peripheral stress responses. Strategies may include minimally invasive surgical techniques to reduce tissue injury, multimodal analgesia to decrease opioid exposure and related immunomodulatory effects, and the use of anesthetic agents with potential organ-protective properties, such as dexmedetomidine, to attenuate neuroinflammatory responses (Kotekar et al., 2014; Su et al., 2016).

At the interaction interfaces (blocking pathological convergence), therapeutic strategies should target key biological mediators involved in pathway integration. Current translational research is exploring BBB protectants and modulators of microglial polarization, such as minocycline, with the aim of interrupting the pathological convergence between central vulnerability and peripheral stress amplification (Subramaniyan and Terrando, 2019).

### 5.3 Unresolved questions and future perspectives

Although the dual-pathway framework provides an integrated perspective for POCD research, several important questions remain unresolved. First, the causal relationships among the major components of the two pathways require further validation. Future investigations should combine multimodal imaging techniques, including positron emission tomography-magnetic resonance imaging, with high-dimensional multi-omics analyses to clarify the temporal and mechanistic relationships among vulnerability characteristics, stress-signal amplification, and cognitive decline (Deschamps et al., 2016; Kline et al., 2012).

Second, the relationship between POCD and long-term postoperative cognitive disorders, including Alzheimer’s disease and related dementias, remains incompletely understood (Evered et al., 2018; Kline et al., 2012). Risk prediction models based on the dual-pathway framework may help address this issue by integrating multidimensional perioperative data, including preoperative vulnerability profiles, perioperative stress exposure, and responses at key interaction interfaces (Ashton et al., 2019; Evered et al., 2018; Roseborough et al., 2023). Such models may improve prediction of the transition from transient POCD to persistent cognitive impairment and facilitate earlier intervention. Progress in these areas may help shift POCD management from an experience-based clinical approach toward a mechanism-driven precision medicine paradigm.

## 6 Summary

Postoperative cognitive dysfunction in geriatric trauma patients is not caused by a single factor but results from the interaction between intrinsic vulnerability and extrinsic perioperative stress within the biological context of aging. Intrinsic vulnerability is primarily reflected in central nervous system susceptibility, including structural brain aging, accumulation of pathological comorbidities, and depletion of cognitive reserve. Extrinsic stressors involve peripheral stress amplification, including trauma-related injury, the neuroimmunomodulatory effects of anesthesia and surgery, and dysregulation of perioperative stress–immune–metabolic networks. These processes converge at key biological interfaces, including the BBB, microglia, and the autonomic nervous system, ultimately contributing to cognitive decline.

From a future clinical perspective, the dual-pathway framework may support a paradigm shift in perioperative neurocognitive protection. Rather than focusing solely on anesthetic management, this model advocates an integrated strategy that combines brain health preservation with regulation of perioperative stress responses throughout the surgical period. Along the temporal dimension, this approach emphasizes the coordination of preoperative vulnerability assessment, intraoperative stress modulation, and postoperative rehabilitation. Along the mechanistic dimension, it targets both enhancement of central resilience and reduction of peripheral insult. Together, these concepts provide a theoretical foundation for individualized diagnostic and therapeutic strategies aimed at the precise prevention and management of POCD.

## Data Availability

The original contributions presented in the study are included in this article/Supplementary material, further inquiries can be directed to the corresponding authors.

## References

[B1] Alam A. Hana Z. Jin Z. Suen K. C. Ma D. (2018). Surgery, neuroinflammation and cognitive impairment. *EBioMedicine* 37 547–556. 10.1016/j.ebiom.2018.10.021 30348620 PMC6284418

[B2] Albanese A. M. Ramazani N. Greene N. Bruse L. (2022). Review of postoperative delirium in geriatric patients after hip fracture treatment. *Geriatr. Orthop. Surg. Rehabil.* 13:21514593211058947. 10.1177/21514593211058947 35282299 PMC8915233

[B3] Arefayne N. R. Berhe Y. W. van Zundert A. A. (2023). Incidence and factors related to prolonged Postoperative Cognitive Decline (POCD) in elderly patients following surgery and anaesthesia: A systematic review. *J. Multidiscip. Healthc.* 16 3405–3413. 10.2147/JMDH.S431168 37964799 PMC10642348

[B4] Ashton N. J. Leuzy A. Lim Y. M. Troakes C. Hortobágyi T. Höglund K. et al. (2019). Increased plasma neurofilament light chain concentration correlates with severity of post-mortem neurofibrillary tangle pathology and neurodegeneration. *Acta Neuropathol. Commun.* 7:5. 10.1186/s40478-018-0649-3 30626432 PMC6327431

[B5] Banks W. A. Reed M. J. Logsdon A. F. Rhea E. M. Erickson M. A. (2021). Healthy aging and the blood-brain barrier. *Nat. Aging* 1 243–254. 10.1038/s43587-021-00043-5 34368785 PMC8340949

[B6] Berger M. Nadler J. W. Browndyke J. Terrando N. Ponnusamy V. Cohen H. J. et al. (2015). Postoperative cognitive dysfunction: Minding the gaps in our knowledge of a common postoperative complication in the elderly. *Anesthesiol. Clin.* 33 517–550. 10.1016/j.anclin.2015.05.008 26315636 PMC4555995

[B7] Bowman G. L. Dayon L. Kirkland R. Wojcik J. Peyratout G. Severin I. C. et al. (2018). Blood-brain barrier breakdown, neuroinflammation, and cognitive decline in older adults. *Alzheimers Dement.* 14 1640–1650. 10.1016/j.jalz.2018.06.2857 30120040

[B8] Cabeza R. Albert M. Belleville S. Craik F. I. M. Duarte A. Grady C. L. et al. (2018). Maintenance, reserve and compensation: The cognitive neuroscience of healthy ageing. *Nat. Rev. Neurosci.* 19 701–710. 10.1038/s41583-018-0068-2 30305711 PMC6472256

[B9] Chan M. Y. Park D. C. Savalia N. K. Petersen S. E. Wig G. S. (2014). Decreased segregation of brain systems across the healthy adult lifespan. *Proc. Natl. Acad. Sci. U. S. A.* 111 E4997–E5006. 10.1073/pnas.1415122111 25368199 PMC4246293

[B10] Chen K. Du X. Chao M. A. Xie Z. Yang G. (2025). Surgery impairs glymphatic activity and cognitive function in aged mice. *Mol. Brain* 18:7. 10.1186/s13041-025-01177-y 39856767 PMC11763125

[B11] Cibelli M. Fidalgo A. R. Terrando N. Ma D. Monaco C. Feldmann M. et al. (2010). Role of interleukin-1beta in postoperative cognitive dysfunction. *Ann. Neurol.* 68 360–368. 10.1002/ana.22082 20818791 PMC4836445

[B12] Cunningham C. (2013). Microglia and neurodegeneration: The role of systemic inflammation. *Glia* 61 71–90. 10.1002/glia.22350 22674585

[B13] Damoiseaux J. S. Beckmann C. F. Arigita E. J. Barkhof F. Scheltens P. Stam C. J. et al. (2008). Reduced resting-state brain activity in the “default network” in normal aging. *Cereb. Cortex* 18 1856–1864. 10.1093/cercor/bhm207 18063564

[B14] Deiner S. Silverstein J. H. (2009). Postoperative delirium and cognitive dysfunction. *Br. J. Anaesth.* 103 (Suppl. 1), i41–i46. 10.1093/bja/aep291 20007989 PMC2791855

[B15] Desborough J. P. (2000). The stress response to trauma and surgery. *Br. J. Anaesth.* 85 109–117. 10.1093/bja/85.1.109 10927999

[B16] Deschamps A. Hall R. Grocott H. Mazer C. D. Choi P. T. Turgeon A. F. et al. (2016). Cerebral oximetry monitoring to maintain normal cerebral oxygen saturation during high-risk cardiac surgery: A randomized controlled feasibility trial. *Anesthesiology* 124 826–836. 10.1097/ALN.0000000000001029 26808629

[B17] Dobson G. P. Morris J. L. Letson H. L. (2022). Immune dysfunction following severe trauma: A systems failure from the central nervous system to mitochondria. *Front. Med.* 9:968453. 10.3389/fmed.2022.968453 36111108 PMC9468749

[B18] Echeverria V. Mendoza C. Iarkov A. (2023). Nicotinic acetylcholine receptors and learning and memory deficits in Neuroinflammatory diseases. *Front. Neurosci.* 17:1179611. 10.3389/fnins.2023.1179611 37255751 PMC10225599

[B19] Erickson M. A. Banks W. A. (2019). Age-Associated changes in the immune system and blood–brain barrier functions. *Int. J. Mol. Sci.* 20:1632. 10.3390/ijms20071632 30986918 PMC6479894

[B20] Evered L. A. Silbert B. S. (2018). Postoperative cognitive dysfunction and noncardiac surgery. *Anesth. Analg* 127 496–505. 10.1213/ANE.0000000000003514 29889707

[B21] Evered L. Silbert B. Knopman D. S. Scott D. A. DeKosky S. T. Rasmussen L. S. et al. (2018). Recommendations for the nomenclature of cognitive change associated with anaesthesia and surgery-2018. *Br. J. Anaesth.* 121 1005–1012. 10.1016/j.bja.2017.11.087 30336844 PMC7069032

[B22] Feinkohl I. Winterer G. Spies C. D. Pischon T. (2017). Cognitive reserve and the risk of postoperative cognitive dysfunction. *Dtsch. Arztebl. Int.* 114 110–117. 10.3238/arztebl.2017.0110 28302254 PMC5359463

[B23] Habes M. Erus G. Toledo J. B. Zhang T. Bryan N. Launer L. J. et al. (2016). White matter hyperintensities and imaging patterns of brain ageing in the general population. *Brain* 139(Pt 4), 1164–1179. 10.1093/brain/aww008 26912649 PMC5006227

[B24] He H. J. Wang Y. Le Y. Duan K. M. Yan X. B. Liao Q. et al. (2012). Surgery upregulates high mobility group box-1 and disrupts the blood-brain barrier causing cognitive dysfunction in aged rats. *CNS Neurosci. Ther.* 18 994–1002. 10.1111/cns.12018 23078219 PMC6493557

[B25] Hergert D. C. Gaasedelen O. Ryman S. G. Prestopnik J. Caprihan A. Rosenberg G. A. (2024). Blood-Brain barrier permeability is associated with cognitive functioning in normal aging and neurodegenerative diseases. *J. Am. Heart Assoc.* 13:e034225. 10.1161/JAHA.124.034225 38979810 PMC11292768

[B26] Horan R. Sortica da Costa C. Nambyiah P. (2023). The persistent effects of anaesthesia on the brain. *BJA Educ.* 23 304–311. 10.1016/j.bjae.2023.04.001 37465234 PMC10350555

[B27] Humeidan M. L. Reyes J. C. Mavarez-Martinez A. Roeth C. Nguyen C. M. Sheridan E. et al. (2021). Effect of cognitive prehabilitation on the incidence of postoperative delirium among older adults undergoing major noncardiac surgery: The neurobics randomized clinical trial. *JAMA Surg.* 156 148–156. 10.1001/jamasurg.2020.4371 33175114 PMC7658803

[B28] Hussain M. Berger M. Eckenhoff R. G. Seitz D. P. (2014). General anesthetic and the risk of dementia in elderly patients: Current insights. *Clin. Interv. Aging* 9 1619–1628. 10.2147/CIA.S49680 25284995 PMC4181446

[B29] Iadecola C. (2013). The pathobiology of vascular dementia. *Neuron* 80 844–866. 10.1016/j.neuron.2013.10.008 24267647 PMC3842016

[B30] Ji R. R. Nackley A. Huh Y. Terrando N. Maixner W. (2018). Neuroinflammation and central sensitization in chronic and widespread pain. *Anesthesiology* 129 343–366. 10.1097/ALN.0000000000002130 29462012 PMC6051899

[B31] Jia S. Yang H. Huang F. Fan W. (2023). Systemic inflammation, neuroinflammation and perioperative neurocognitive disorders. *Inflamm. Res.* 72 1895–1907. 10.1007/s00011-023-01792-2 37688642

[B32] Jiang Y. Yabluchanskiy A. Deng J. Amil F. A. Po S. S. Dasari T. W. (2022). The role of age-associated autonomic dysfunction in inflammation and endothelial dysfunction. *Geroscience* 44 2655–2670. 10.1007/s11357-022-00616-1 35773441 PMC9768093

[B33] Karagianni M. Brotis A. G. Vrettou C. S. Goupou K. Stranjalis G. Fountas K. N. (2025). Cerebral perfusion pressure in severe traumatic brain injury survivors and non-survivors: A meta-analysis. *Brain Sci.* 15:1161. 10.3390/brainsci15111161 41300168 PMC12649846

[B34] Khaled M. Sabac D. Fuda M. Koubaesh C. Gallab J. Qu M. et al. (2025). Postoperative pain and neurocognitive outcomes after noncardiac surgery: A systematic review and dose-response meta-analysis. *Br. J. Anaesth.* 134 89–101. 10.1016/j.bja.2024.08.032 39393998

[B35] Kitthanyateerakul P. Tankumpuan T. Davidson P. M. (2024). Cognitive dysfunction in older patients undergoing non-neurosurgery in the immediate postoperative period: A systematic review. *Nurs. Open* 11:e70023. 10.1002/nop2.70023 39189543 PMC11348231

[B36] Kline R. P. Pirraglia E. Cheng H. De Santi S. Li Y. Haile M. et al. (2012). Surgery and brain atrophy in cognitively normal elderly subjects and subjects diagnosed with mild cognitive impairment. *Anesthesiology* 116 603–612. 10.1097/ALN.0b013e318246ec0b 22293721 PMC3418798

[B37] Kong E. Geng X. Wu F. Yue W. Sun Y. Feng X. (2024). Microglial exosome miR-124-3p in hippocampus alleviates cognitive impairment induced by postoperative pain in elderly mice. *J. Cell Mol. Med.* 28 e18090. 10.1111/jcmm.18090 38140846 PMC10844686

[B38] Kotekar N. Kuruvilla C. S. Murthy V. (2014). Post-operative cognitive dysfunction in the elderly: A prospective clinical study. *Indian J. Anaesth.* 58 263–268. 10.4103/0019-5049.135034 25024467 PMC4090990

[B39] Liu H. Yang Y. Xia Y. Zhu W. Leak R. K. Wei Z. et al. (2017). Aging of cerebral white matter. *Ageing Res. Rev.* 34 64–76. 10.1016/j.arr.2016.11.006 27865980 PMC5250573

[B40] Liu Y. Fu H. Wang T. (2022). Neuroinflammation in perioperative neurocognitive disorders: From bench to the bedside. *CNS Neurosci. Ther.* 28 484–496. 10.1111/cns.13794 34990087 PMC8928922

[B41] Liu Y. Liu Q. Wang H. Qiu Y. Lin J. Wu W. et al. (2024). Hippocampal synaptic plasticity injury mediated by SIRT1 downregulation is involved in chronic pain-related cognitive dysfunction. *CNS Neurosci. Ther.* 30 e14410. 10.1111/cns.14410 PMC10848102 37592394

[B42] Lord J. M. Midwinter M. J. Chen Y. F. Belli A. Brohi K. Kovacs E. J. et al. (2014). The systemic immune response to trauma: An overview of pathophysiology and treatment. *Lancet* 384 1455–1465. 10.1016/S0140-6736(14)60687-5 25390327 PMC4729362

[B43] McEwen B. S. (1998). Protective and damaging effects of stress mediators. *N. Engl. J. Med.* 338 171–179. 10.1056/NEJM199801153380307 9428819

[B44] Meng Q. Su S. Lei L. Zhang Y. Duan J. Ren X. et al. (2025). CHOP-Mediated disruption of hippocampal synaptic plasticity and neuronal activity contributes to chronic pain-related cognitive deficits. *CNS Neurosci. Ther.* 31:e70160. 10.1111/cns.70160 39817595 PMC11736631

[B45] Montagne A. Barnes S. R. Sweeney M. D. Halliday M. R. Sagare A. P. Zhao Z. et al. (2015). Blood-brain barrier breakdown in the aging human hippocampus. *Neuron* 85 296–302. 10.1016/j.neuron.2014.12.032 25611508 PMC4350773

[B46] Newfield P. (2009). Postoperative cognitive dysfunction. *F1000 Med. Rep.* 1:14. 10.3410/M1-14 20948768 PMC2920713

[B47] Nilsson U. Amirpour A. Lampi M. Guenna Holmgren A. Markovic G. Zecevic E. et al. (2025). Older patients’ postoperative neurocognitive recovery: A narrative review. *Clin. Interv. Aging* 20 2579–2591. 10.2147/CIA.S559531 41425754 PMC12717814

[B48] Olivieri F. Biscetti L. Pimpini L. Pelliccioni G. Sabbatinelli J. Giunta S. (2024). Heart rate variability and autonomic nervous system imbalance: Potential biomarkers and detectable hallmarks of aging and inflammaging. *Ageing Res. Rev.* 101:102521. 10.1016/j.arr.2024.102521 39341508

[B49] Osman V. Speigel I. Patel K. Hemmings H. C. Jr. (2023). Isoflurane alters presynaptic endoplasmic reticulum calcium dynamics in wild-type and malignant hyperthermia-susceptible rodent hippocampal neurons. *eNeuro* 10:ENEURO.0114-23.2023. 10.1523/ENEURO.0114-23.2023 37591734 PMC10467020

[B50] Pavlov V. A. Tracey K. J. (2012). The vagus nerve and the inflammatory reflex–linking immunity and metabolism. *Nat. Rev. Endocrinol.* 8 743–754. 10.1038/nrendo.2012.189 23169440 PMC4082307

[B51] Perry V. H. Holmes C. (2014). Microglial priming in neurodegenerative disease. *Nat. Rev. Neurol.* 10 217–224. 10.1038/nrneurol.2014.38 24638131

[B52] Qin M. N. Deng Y. N. (2026). Anesthesia-related factors in the pathogenesis of postoperative cognitive dysfunction: A mechanistic perspective. *Front. Neurol.* 16:1700911. 10.3389/fneur.2025.1700911 41561335 PMC12812579

[B53] Ren X. Huiqiao L. Wu Y. Zhang T. Chen P. Li L. et al. (2025). Perioperative neurocognitive disorders: A comprehensive review of terminology, clinical implications, and future research directions. *Front. Neurol.* 16:1526021. 10.3389/fneur.2025.1526021 40933055 PMC12418783

[B54] Roseborough A. D. Saad L. Goodman M. Cipriano L. E. Hachinski V. C. Whitehead S. N. (2023). White matter hyperintensities and longitudinal cognitive decline in cognitively normal populations and across diagnostic categories: A meta-analysis, systematic review, and recommendations for future study harmonization. *Alzheimers Dement.* 19 194–207. 10.1002/alz.12642 35319162

[B55] Rundshagen I. (2014). Postoperative cognitive dysfunction. *Dtsch. Arztebl. Int.* 111 119–125. 10.3238/arztebl.2014.0119 24622758 PMC3959222

[B56] Sacerdote P. (2006). Opioids and the immune system. *Palliat. Med.* 20 (Suppl. 1), s9–s15. 10.1191/0269216306pm1124oa 16764216

[B57] Safavynia S. A. Goldstein P. A. (2019). The role of neuroinflammation in postoperative cognitive dysfunction: Moving from hypothesis to treatment. *Front. Psychiatry* 9:752. 10.3389/fpsyt.2018.00752 30705643 PMC6345198

[B58] Saxena S. Kruys V. De Jongh R. Vamecq J. Maze M. (2021). High-Mobility group Box-1 and its potential role in perioperative neurocognitive disorders. *Cells* 10:2582. 10.3390/cells10102582 34685561 PMC8533835

[B59] Seplovich G. Bouchi Y. de Rivero Vaccari J. P. Pareja J. C. M. Reisner A. Blackwell L. et al. (2025). Inflammasome links traumatic brain injury, chronic traumatic encephalopathy, and Alzheimer’s disease. *Neural. Regen. Res.* 20 1644–1664. 10.4103/NRR.NRR-D-24-00107 39104096 PMC11688549

[B60] Silbert B. Evered L. Scott D. A. McMahon S. Choong P. Ames D. et al. (2015). Preexisting cognitive impairment is associated with postoperative cognitive dysfunction after hip joint replacement surgery. *Anesthesiology* 122 1224–1234. 10.1097/ALN.0000000000000671 25859906

[B61] Skvarc D. R. Berk M. Byrne L. K. Dean O. M. Dodd S. Lewis M. et al. (2018). Post-Operative cognitive dysfunction: An exploration of the inflammatory hypothesis and novel therapies. *Neurosci. Biobehav. Rev.* 84 116–133. 10.1016/j.neubiorev.2017.11.011 29180259

[B62] Stern Y. (2012). Cognitive reserve in ageing and Alzheimer’s disease. *Lancet Neurol.* 11 1006–1012. 10.1016/S1474-4422(12)70191-6 23079557 PMC3507991

[B63] Su X. Meng Z. T. Wu X. H. Cui F. Li H. L. Wang D. X. et al. (2016). Dexmedetomidine for prevention of delirium in elderly patients after non-cardiac surgery: A randomised, double-blind, placebo-controlled trial. *Lancet* 388 1893–1902. 10.1016/S0140-6736(16)30580-3 27542303

[B64] Subramaniyan S. Terrando N. (2019). Neuroinflammation and perioperative neurocognitive disorders. *Anesth. Analg.* 128 781–788. 10.1213/ANE.0000000000004053 30883423 PMC6437083

[B65] Suraarunsumrit P. Srinonprasert V. Kongmalai T. Suratewat S. Chaikledkaew U. Rattanasiri S. et al. (2024). Outcomes associated with postoperative cognitive dysfunction: A systematic review and meta-analysis. *Age Ageing* 53:afae160. 10.1093/ageing/afae160 39058915 PMC11277860

[B66] Sweeney M. D. Kisler K. Montagne A. Toga A. W. Zlokovic B. V. (2018). The role of brain vasculature in neurodegenerative disorders. *Nat. Neurosci.* 21 1318–1331. 10.1038/s41593-018-0234-x 30250261 PMC6198802

[B67] Tang N. Duan J. Liu X. Li P. Hao X. Zhang L. (2025). Developed a knowledge base of risk factors for postoperative cognitive dysfunction: A retrospective database study. *Sci. Rep.* 15:21004. 10.1038/s41598-025-07266-1 40594746 PMC12214832

[B68] Terrando N. Brzezinski M. Degos V. Eriksson L. I. Kramer J. H. Leung J. M. et al. (2011). Perioperative cognitive decline in the aging population. *Mayo Clin. Proc.* 86 885–893. 10.4065/mcp.2011.0332 21878601 PMC3257991

[B69] Terrando N. Monaco C. Ma D. Foxwell B. M. Feldmann M. Maze M. (2010). Tumor necrosis factor-alpha triggers a cytokine cascade yielding postoperative cognitive decline. *Proc. Natl. Acad. Sci. U. S. A.* 107 20518–20522. 10.1073/pnas.1014557107 21041647 PMC2996666

[B70] Terrando N. Yang T. Wang X. Fang J. Cao M. Andersson U. et al. (2016). Systemic HMGB1 neutralization prevents postoperative neurocognitive dysfunction in aged rats. *Front. Immunol.* 7:441. 10.3389/fimmu.2016.00441 27822212 PMC5075578

[B71] Thayer J. F. Yamamoto S. S. Brosschot J. F. (2010). The relationship of autonomic imbalance, heart rate variability and cardiovascular disease risk factors. *Int. J. Cardiol.* 141 122–131. 10.1016/j.ijcard.2009.09.543 19910061

[B72] Tracey K. J. (2002). The inflammatory reflex. *Nature* 420 853–859. 10.1038/nature01321 12490958

[B73] Vacas S. Degos V. Feng X. Maze M. (2013). The neuroinflammatory response of postoperative cognitive decline. *Br. Med. Bull.* 106 161–178. 10.1093/bmb/ldt006 23558082 PMC4990823

[B74] Varatharaj A. Galea I. (2017). The blood-brain barrier in systemic inflammation. *Brain Behav. Immun.* 60 1–12. 10.1016/j.bbi.2016.03.010 26995317

[B75] Wang P. Velagapudi R. Kong C. Rodriguiz R. M. Wetsel W. C. Yang T. et al. (2020). Neurovascular and immune mechanisms that regulate postoperative delirium superimposed on dementia. *Alzheimers Dement.* 16 734–749. 10.1002/alz.12064 32291962 PMC7317948

[B76] Wu M. Zhang M. Yin X. Chen K. Hu Z. Zhou Q. et al. (2021). The role of pathological tau in synaptic dysfunction in Alzheimer’s diseases. *Transl. Neurodegener.* 10:45. 10.1186/s40035-021-00270-1 34753506 PMC8579533

[B77] Wu X. He T. He F. Liu L. (2025). Is postoperative cognitive dysfunction a disease of microglial inflammatory memory? A state-transition model from metabolic stress to epigenetic lock-in. *Front. Mol. Neurosci.* 18:1648161. 10.3389/fnmol.2025.1648161 40843245 PMC12364888

[B78] Wu Y. Yu C. Gao F. (2023). Risk factors for postoperative cognitive dysfunction in elderly patients undergoing surgery for oral malignancies. *Perioper. Med.* 12:42. 10.1186/s13741-023-00330-2 37468994 PMC10357604

[B79] Yang S. Gu C. Mandeville E. T. Dong Y. Esposito E. Zhang Y. et al. (2017). Anesthesia and surgery impair blood-brain barrier and cognitive function in mice. *Front. Immunol.* 8:902. 10.3389/fimmu.2017.00902 28848542 PMC5552714

[B80] Yirmiya R. Goshen I. (2011). Immune modulation of learning, memory, neural plasticity and neurogenesis. *Brain Behav. Immun.* 25 181–213. 10.1016/j.bbi.2010.10.015 20970492

[B81] Yu L. Wen G. Zhu S. Hu X. Huang C. Yang Y. (2021). Abnormal phosphorylation of tau protein and neuroinflammation induced by laparotomy in an animal model of postoperative delirium. *Exp. Brain Res.* 239 867–880. 10.1007/s00221-020-06007-2 33409674

[B82] Zhang Y. Ma R. Wang W. Deng Q. Cao C. Yu C. et al. (2023). Activation of α7nAChR by PNU282987 improves cognitive impairment through inhibiting oxidative stress and neuroinflammation in D-galactose induced aging via regulating α7nAChR/Nrf2/HO-1 signaling pathway. *Exp. Gerontol.* 175:112139. 10.1016/j.exger.2023.112139 36898594

[B83] Zhao Q. Wan H. Pan H. Xu Y. (2024). Postoperative cognitive dysfunction-current research progress. *Front. Behav. Neurosci.* 18:1328790. 10.3389/fnbeh.2024.1328790 38357422 PMC10865506

[B84] Zorbaz T. Madrer N. Soreq H. (2022). Cholinergic blockade of neuroinflammation: From tissue to RNA regulators. *Neuronal Signal.* 6:NS20210035. 10.1042/NS20210035 35211331 PMC8837817

