# Supplementary material for: Risk factors for postoperative cognitive dysfunction in geriatric trauma: a dual-pathway perspective
Source: Front Aging Neurosci. 2026 Jul 17;18:1841750. doi: 10.3389/fnagi.2026.1841750 (PMC13423957; doi:10.3389/fnagi.2026.1841750)
Supplement: Supplementary file 1 [file Table_1.docx]

**Supplementary Table 1. Summary of the Dual-Pathway Framework for POCD in Geriatric Trauma**

| **Component** | **Pathway I: central vulnerability** | **Pathway II: peripheral stress amplification** |
| --- | --- | --- |
| Core nature | Endogenous susceptibility | Exogenous triggers |
| Key factors | Brain aging, neurodegeneration, reduced cognitive reserve | Trauma, surgery, anesthesia, systemic inflammation |
| Biological features | BBB dysfunction, microglial priming, impaired neural networks | Hemodynamic instability, inflammatory cytokines, neuroendocrine stress |
| Representative markers | WMH, hippocampal atrophy, NfL, GFAP | IL-6, CRP, cortisol, rSO₂ |
| Functional impact | Lower cognitive threshold | Increased stress burden |
| Clinical implication | Identify high-risk patients | Monitor perioperative stress |
| Intervention focus | Enhance resilience (prehabilitation, vascular control) | Reduce insults (analgesia, minimally invasive surgery) |
| Interaction interface | BBB, microglia, autonomic system | BBB, microglia, autonomic system |

**Abbreviations**: POCD, postoperative cognitive dysfunction; WMH, white matter hyperintensities; NfL, neurofilament light chain; GFAP, glial fibrillary acidic protein; IL-6, interleukin-6; CRP, C-reactive protein; rSO₂, regional cerebral oxygen saturation; BBB, blood–brain barrier.
